# Preoperative ultrasound to identify prefrailty or mild cognitive impairment in patients undergoing total hip/knee replacement: a single-center, prospective, cohort pilot study

**DOI:** 10.3389/fmed.2026.1878659

**Published:** 2026-09-25

**Authors:** Heng Xue, Meng Kang, Jiuping Huang, Xiaoxiao Wang, Xuan Lai, Zhe Ma, Qian Xiang, Hua Tian, Xiangyang Guo, Yang Li, Yongzheng Han

**Affiliations:** 1 Department of Ultrasound, Peking University Third Hospital, Beijing, China; 2 Department of Anesthesiology, Peking University Third Hospital, Beijing, China; 3 Research Center of Clinical Epidemiology, Peking University Third Hospital, Beijing, China; 4 Geriatrics Department, Peking University Third Hospital, Beijing, China; 5 Peking University Health Science Center, Beijing, China; 6 Department of Orthopedics, Peking University Third Hospital, Beijing, China; 7 Engineering Research Center of Bone and Joint Precision Medicine, Ministry of Education, Beijing, China

**Keywords:** mild cognitive impairment, muscle stiffness, muscle thickness, prefrailty, shear wave elastography, ultrasound

## Abstract

**Objectives:**

To identify prefrailty and mild cognitive impairment (MCI) in patients undergoing total hip/knee replacement using preoperative ultrasound measurements of muscle dimensions and stiffness.

**Methods:**

This pilot study was conducted at a large tertiary public hospital in Beijing, China, and included 121 consecutive patients with hip or knee osteoarthritis scheduled for total hip or knee replacement [median age 71 years, interquartile range (IQR) 5 years; 94 women, 77.7%]. Preoperative assessments included measurements of the dimensions and stiffness of the biceps brachii (BB) using grey scale ultrasound (US) and shear wave elastography (SWE). Patients were preoperatively assessed for prefrailty and mild cognitive impairment (MCI) based on FRAIL scale and Montreal Cognitive Assessment (MoCA) combined with the Clinical Dementia Rating Scale (CDR). Prospective subgroup analyses stratified by gender were performed to identify parameters with statistically significant differences between the non-prefrail and prefrail groups, as well as between the non-MCI and MCI groups.

**Results:**

Subgroup analysis by gender showed that thickness of BB on both dominant and non-dominant side in prefrail group from male patients were significantly lower than that in non-frail group. The thickness of the BB on the non-dominant side in predicting prefrailty among male patients had the highest AUC (0.824, 95% CI: 0.627–1.000), with an optimal cut-off value of 1.85 cm, achieving sensitivity of 77.8%, specificity of 66.7%. However, this finding is exploratory given the small male prefrail subgroup (*n* = 9) and requires validation in adequately powered cohorts. In female participants, no parameters showed a significant difference between the non-prefrail and prefrail groups. Additionally, no significant differences were observed in ultrasound measurements between MCI and non-MCI groups.

**Conclusion:**

In this pilot study, preoperative US measurements of BB thickness demonstrated preliminary promise for predicting prefrailty among male patients undergoing total hip/knee replacement. These findings are hypothesis-generating and require confirmation in larger, independent cohorts. However, this approach showed limited utility in female participants and was not effective in predicting MCI.

**Clinical Trial Registration**: https:// www.chictr.org.cn/showproj.aspx?proj=177048, identifier ChiCTR2200062483.

## Background

Frailty is defined as increased vulnerability and impaired recovery from stressors, such as falls and infections, and is also associated with premature mortality and prolonged hospitalization (1, 2). Mild cognitive impairment (MCI) is defined by memory impairment and other cognitive dysfunctions, and shares similar adverse health outcomes with frailty (3, 4). The etiology of these two conditions is multifactorial. Beyond physical phenotype, dynamic clinical stressors such as the anticholinergic medication burden have been heavily associated with multidimensional vulnerability in hospitalized oldest-old patients, complicating perioperative stratification (5). Identifying high-risk patients with prefrailty allows for early intervention to prevent adverse outcomes following surgery, thereby reducing the associated morbidity and mortality (6, 7). However, comprehensive assessment of prefrailty and MCI requires a substantial time and the expertise of experienced psychiatrists or geriatric physicians, making it impractical in the demanding clinical setting of surgical systems.

Numerous studies have been conducted to identify potential risk factors associated with frailty and MCI. Recently, low skeletal muscle mass, or sarcopenia, has emerged as a commonly important factor contributing to both frailty (8, 9) and MCI (10–13). In particular, recent 2026 data emphasize the high prevalence of sarcopenia among the oldest-old hospitalized population, while bedside muscle ultrasound has emerged as a crucial tool to evaluate its direct clinical impact on acute cognitive fluctuations like delirium (14, 15). In addition, shear-wave elastography (SWE) of muscle in both static and dynamic conditions provides additional information related to tissue properties by evaluating tissue elasticity, which may contribute to the diagnosis of sarcopenia (16–18) and the estimation of individual muscle force (19). Therefore, in this pilot study, we hypothesized that preoperative US measurements of biceps brachii (BB) dimensions and stiffness could predict frailty and MCI in patients undergoing total hip/knee replacement, while also exploring the determination of useful cutoff values.

## Materials and methods

### Patients and setting

This study was approved by Peking University Third Hospital Medical Science Research Ethics Committee (IRB00006761-M2022303) on May 24th, 2022, and conformed to the principles of the Declaration of Helsinki. All methods were performed in accordance with relevant guidelines and regulations. Written informed consent was obtained from all participants or their guardians, and the study was registered at the Chinese Clinical Trial Registry (ChiCTR2200062483). Patients with hip/knee osteoarthritis scheduled for total hip/knee replacement were recruited from Peking University Third Hospital (Beijing, China) from April 2023 to December 2023. Patients who declined participation in the study were excluded. Patients with neuromuscular disorders (such as muscular dystrophy, spinal muscular atrophy, amyotrophic lateral sclerosis, or polyneuropathy) were also excluded from the study, based on their medical history and clinical presentation (Figure 1). Additionally, patients with significant symptoms in upper limb were excluded, primarily pain and functional impairment that could lead to decreased activity of the upper limb, as these symptoms could lead to decreased activity of the upper limb, thus affecting dimensions and stiffness of the BB, which were key outcome measurements in this study. The collected basic information included patient demographics such as age, sex, weight, height, body mass index (BMI). To assess the nutritional status of patients prior to surgery, the geriatric nutritional risk index (GNRI) and prognostic nutritional index (PNI) were calculated using corresponding formulas (20, 21).

**Figure 1:**
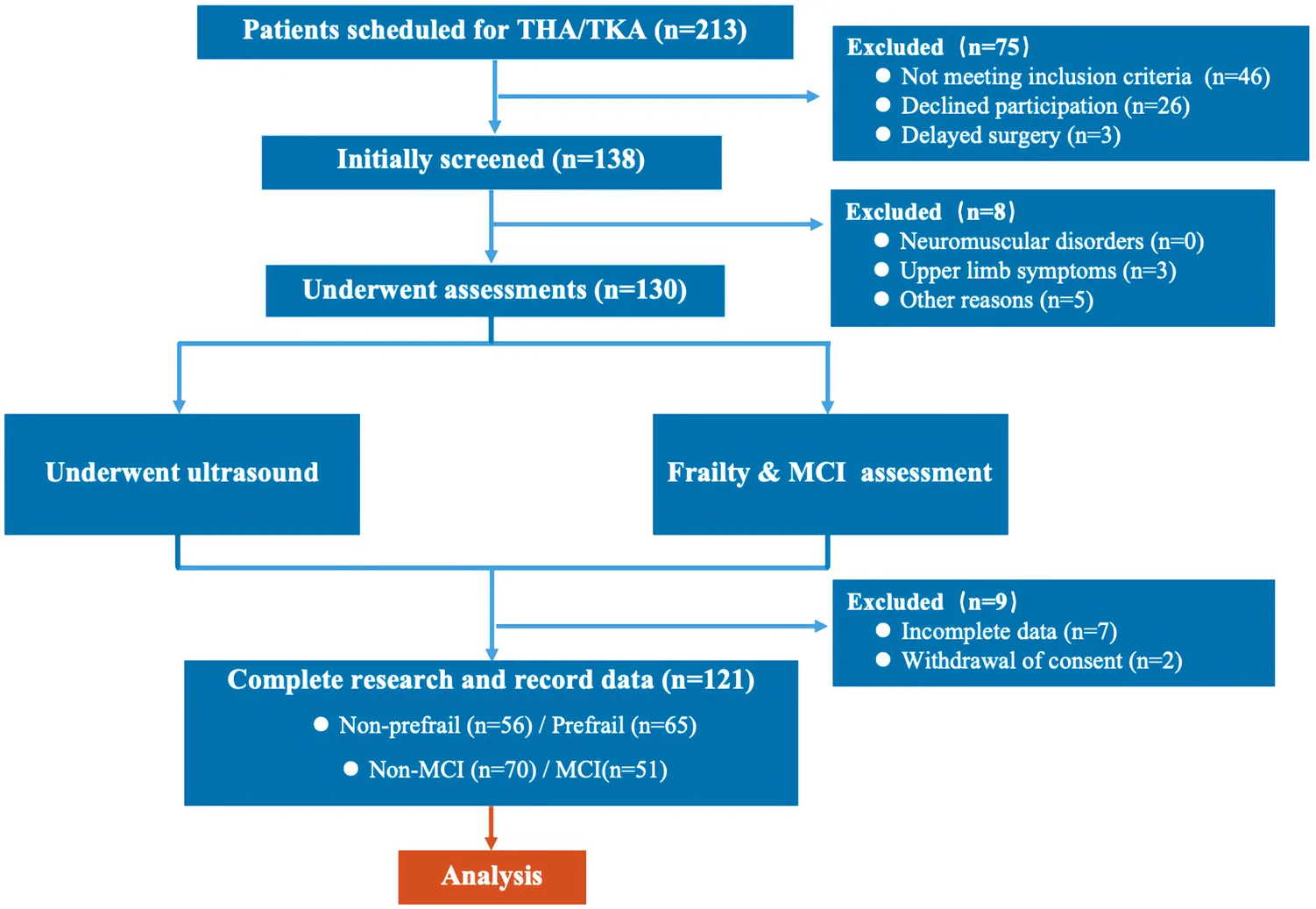
Flow diagram of patient screening, enrollment, and analysis.

### US and shear wave elastography (SWE) examination

US and SWE examinations were performed one day before the surgery by one experienced radiologist with more than 10 years of experience in musculoskeletal US and 5 years of experience in SWE. The intra- and inter-observer reliability of US measurements of BB thickness and SWE-derived stiffness parameters have been previously established as good to excellent in the literature (22, 23).

All grayscale and SWE images and measurements were obtained using a single US system (Aixplorer; SuperSonic Imagine, Aix en Provence, France), equipped with 4–15 and 4–10 MHz liner transducers. The probe was applied to the skin with minimum pressure, since probe compression might affect measurements of muscle dimension. Although the majority of sarcopenia studies based on US have been performed on the quadriceps muscle and/or biceps brachii (24, 25), considering the effect of the primary disease (hip/knee osteoarthritis) on the lower limb muscles, only biliteral biceps brachii (BB) were measured. The patient is seated in front of the examiner with forearm externally rotated to bring the muscle forward (Figure 2A). The muscle was measured in the transverse plane at the midpoint between the coracoid process and the elbow crease. The thickness, perimeter and area of BB muscle were measured on both dominant side and non-dominant side (Figure 3). Muscle thickness was measured as the perpendicular distance from the superficial to the deep fascia. Perimeter and cross-sectional area were measured by manually tracing the border of the muscle using the system's caliper and area measurement tools. Trapezoidal extension may be used when the boundary extended beyond the field of view.

**Figure 2:**
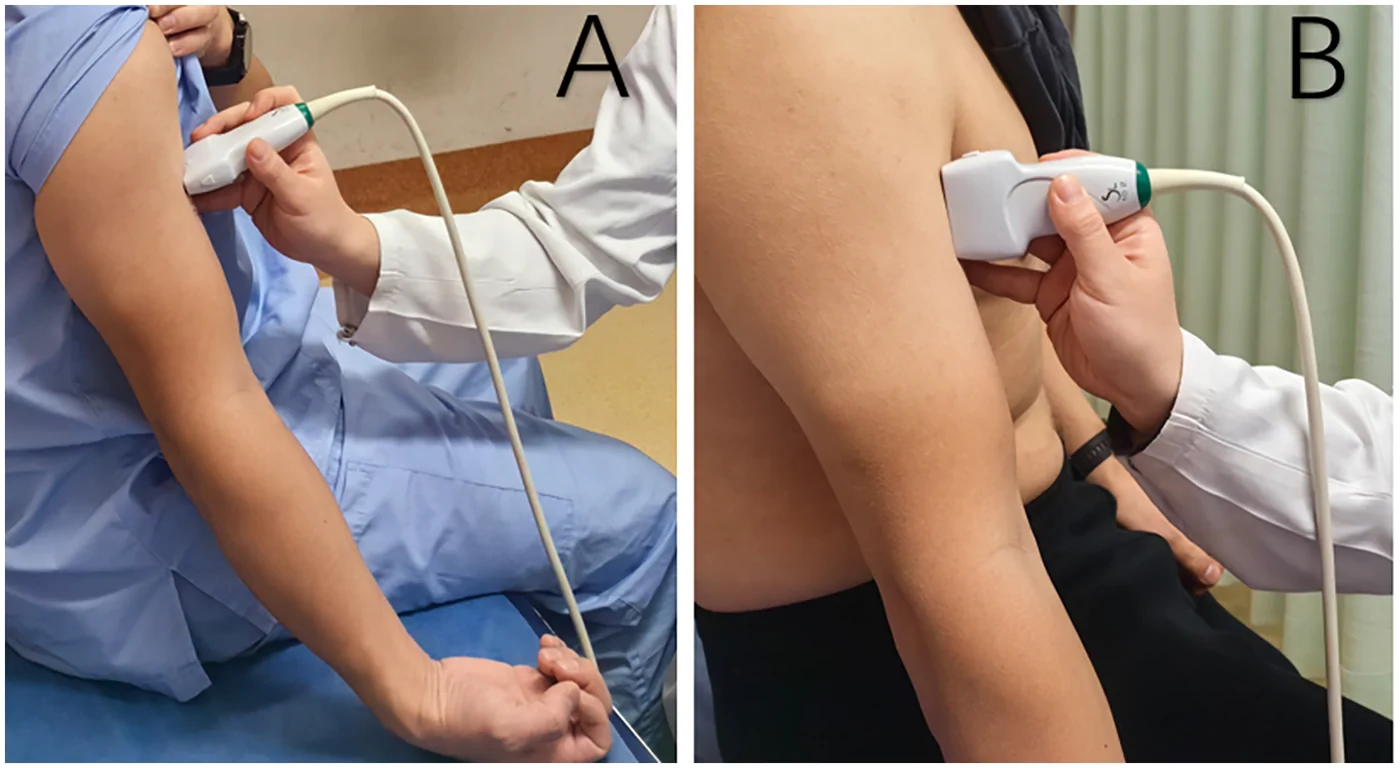
**(A)** Photograph illustrates the patient position and transducer position when measuring the thickness, perimeter and area of BB muscle at transverse plane. **(B)** After grey scale evaluation, SWE was performed at longitudinal plane when the BB muscle is relaxed.

**Figure 3:**
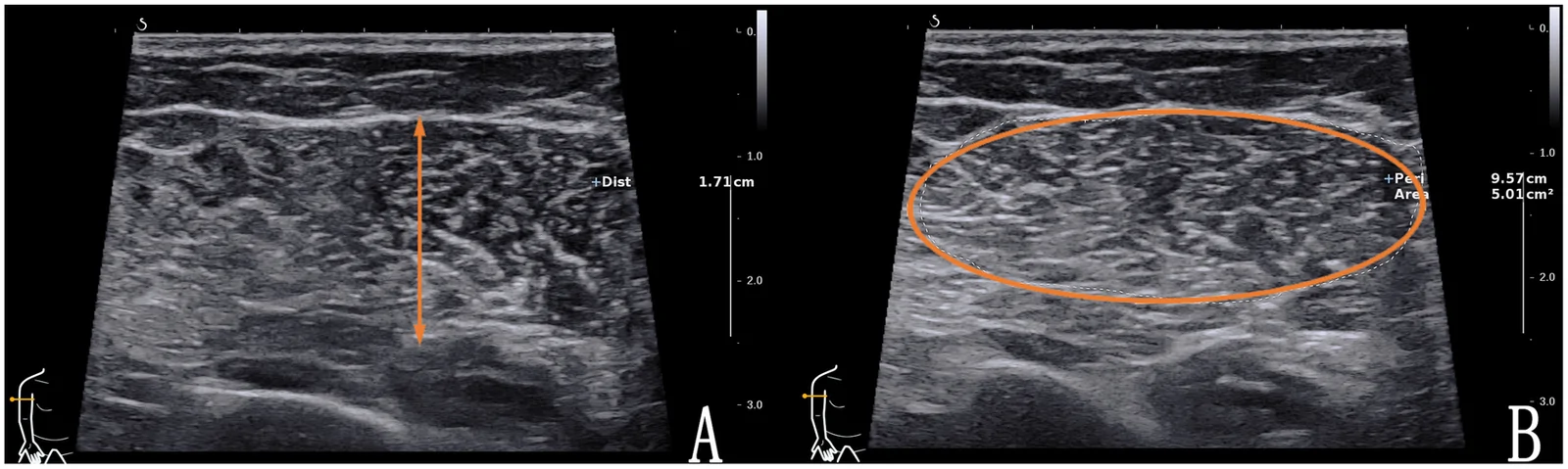
The thickness (**A**, between arrow head), perimeter and area (**B**, orange ellipse) of right BB muscle were measured by grey-scale US. The perimeter and cross-sectional area were obtained by manual tracing of the muscle border.

After grey scale evaluation, SWE was performed by using the 4–10 MHz linear transducer with minimum pressure. The system reports SWE readings in both Shear Wave Velocity (SWV) (meters per second) and Young modulus (kilopascals) as surrogates for tissue stiffness. SWV was used for analysis due to the anisotropy of muscle. Participants were asked to relax the upper limbs, and SWE was performed in the longitudinal plane for its better reproducibility (26) (Figure 2B). During image acquisition, the probe was applied to the skin with minimum pressure and the state immobilization was maintained for several seconds to ensure the acquisition of stable SWE images. The frequency was switched to penetration mode. A rectangular elasticity box was fixed at a size of 2 cm × 1.5 cm, and a circular region of interest (Quantification BOX, Q-BOX) with a fixed diameter of 6 mm was used to calculate muscle elasticity (Figure 4A). SWE images free from random inconsistent artifactual color patterns were selected for measurement. Q-BOX was put at the most homogeneously elastic area of the muscle, away from fascia or bone. Five reliable SWE images were obtained and the median of measurements was used as the result, providing a comparatively unbiased estimation of muscle stiffness. The intra-session variability was assessed by calculating the coefficient of variation, and the overall reproducibility was found to be acceptable (<10%). Elasticity of contracted BB muscle was measured with the same method employed during the relaxed position (Figure 4B).

**Figure 4:**
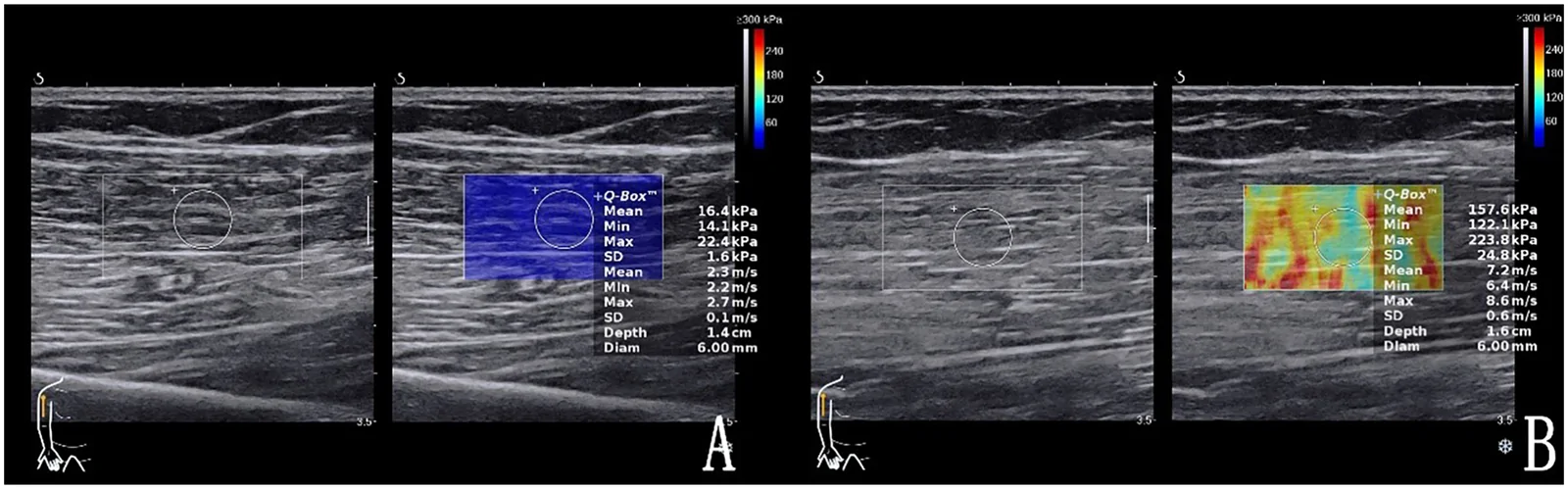
The same patient with Figure 3. Stiffness of BB muscle was measured at longitudinal plane for both relaxed **(A)** and tensed position **(B)** with SWE.

### Prefrailty and MCI assessments

Prefrailty and MCI were assessed by a geriatric physician with over 10 years of experience. Prefrailty was diagnosed using the FRAIL scale (27, 28), which consists of 5 self-report questions. One point is given for any affirmative response, with a total score ranging from 0 to 5 points. Patients with 0 point were assigned to the non-frailty group, while those 1–2 points were assigned to the prefrailty group. The diagnosis of MCI was carried out by senior geriatrician according to the National Institute on Aging Alzheimer's Association workgroups in 2011, with both Montreal Cognitive Assessment (MoCA) <26 (29) and Clinical Dementia Rating Scale (CDR) of 0.5 (30). The assessments of frailty and MCI were conducted after the US and SWE examinations, one day prior to surgery.

### Statistical analysis

The sample size was calculated based on previous research (31) using PASS 11. A random sample of 48 subjects from the prefrail population and 48 subjects from the non-frail population produced a two-sided 95.0% confidence interval with a width of 0.220, assuming a sample AUC (area under the curve) value of 0.650. This calculation was designed for the overall cohort AUC estimation. Gender-stratified analyses were not pre-specified in the sample size calculation and should therefore be considered exploratory, as the male subgroup was insufficiently powered for definitive diagnostic accuracy testing.

We performed statistical analysis using SPSS software (version 25.0, IBM). Categorical variables were described using absolute figures with percentages, while normally distributed continuous variables are expressed as means ± SDs, non-normally distributed continuous variables are presented as medians with IQR. The Shapiro–Wilk test was used to test for normality. Since the thickness and cross-sectional area of the BB muscle are known to differ significantly between males and females (32), subgroup analysis based on gender was performed. To compare the muscle dimensions and stiffness between the prefrail and non-frail group, as well as the MCI and non-MCI groups, Mann–Whitney *U* test was used. Areas under the ROC curves and 95% CIs were calculated in each ROC curve analysis. The optimal cutoff values were determined using the Youden index. *P* < 0.05 was considered indicative of a statistically significant difference.

Of the 130 patients who underwent preoperative assessments, nine were excluded (seven with incomplete data and two withdrawing consent). The remaining 121 patients, all with complete data, were included in the final analysis.

## Results

### Prefrailty and MCI diagnosis in study participants

A total of 121 consecutive patients were included, with a median age of 71 years (IQR 5 years, 94 women). Based on the FRAIL scale, prefrailty was identified in 65 patients (53.7%), and no patients met the criteria for frailty (score ≥ 3). MCI was identified in 51 patients (42.1%). Baseline demographics and nutritional parameters stratified by prefrailty and MCI status are presented in Tables 1A,B, respectively. The proportion of females is higher in the prefrail group compared to the non-frail group. No significant differences were observed in other demographic or nutritional parameters between the two groups.

**Table 1A:** Demographics and clinical parameters between non-frail and prefrail groups.

Parameter	Non-frail (*n* = 56)	Prefrail (*n* = 65)	*P* value
Age, year	70.00 (68.75, 74.00)	70.00 (67.00, 73.00)	0.322
Female (*n*, %)	38 (67.9%)	56 (86.2%)	**0** **.** **016**
BMI	27.2 ± 3.3	27.3 ± 3.3	0.840
GNRI	107.0 ± 3.9	106.5 ± 4.2	0.477
PNI	52.9 ± 4.0	52.6 ± 3.8	0.630
MCI (*n*, %)	24 (42.9%)	27 (41.5%)	0.884

The data in bold represent statistically significant differences.

**Table 1B:** Demographics and clinical parameters between non-MCI and MCI groups.

Parameter	Non-MCI (*n* = 70)	MCI (*n* = 51)	*P* value
Age, year	70.00 (68.00, 73.00)	70.00 (69.00, 73.50)	0.456
Female (*n*, %)	54 (77.1%)	40 (78.4%)	0.867
BMI	26.74 ± 2.86	27.88 ± 3.71	0.070
GNRI	107.13 ± 3.76	106.11 ± 4.42	0.190
PNI	53.18 ± 3.85	52.20 ± 3.92	0.175
Prefrail (*n*, %)	38 (54.3%)	27 (52.9%)	0.884

### Predictive value of ultrasound measurement for prefrailty and MCI

Subgroup analysis by gender showed that thickness of BB on both dominant and non-dominant side in prefrail group from male patients were significantly lower than that in non-frail group. On the dominant side, the median (IQR) thickness was 2.1 cm (0.5 cm) for the non-prefrail group and 1.8 cm (0.5 cm) for the prefrail group (*P* = 0.015). On the non-dominant side, the median (IQR) thickness was 2.2 cm (0.7 cm) for the non-prefrail group and 1.5 cm (0.4 cm) for the prefrail group (*P* = 0.004). Similar result was observed in thickness of BB on non-dominant normalized by BMI (thickness/BMI). No significant differences were found in other parameters, such as muscle perimeter, area or stiffness, between the non-prefrail and prefrail groups (Table 2).

**Table 2:** Difference of ultrasound measurement for non-frail and prefrail group with subgroup analysis by gender.

Parameter	Male			Female		
	Non-frail (*n* = 18)	Prefrail (*n* = 9)	*P* value	Non-frail (*n* = 38)	Prefrail (*n* = 56)	*P* value
Thickness of BB on dominant side, cm	2.1 (0.5)	1.8 (0.5)	**0** **.** **015**	1.7 (0.4)	1.7 (0.4)	0.115
Perimeter of BB on dominant side, cm	13.8 (3.6)	14.6 (4.3)	0.982	11.4 (2.5)	10.8 (2.3)	0.333
Area of BB on dominant side, cm^2^	9.7 (2.5)	8.5 (2.6)	0.145	6.4 (2.3)	6.6 (1.7)	0.135
SWV of BB on dominant side, m/s	2.3 (0.9)	2.1 (0.2)	0.263	2.4 (0.8)	2.3 (0.7)	0.790
Thickness of BB on non-dominant side, cm	2.2 (0.7)	1.5 (0.4)	**0** **.** **004**	1.7 (0.5)	1.7 (0.4)	0.393
Perimeter of BB on non-dominant side, cm	12.8 (3.0)	13.8 (4.7)	0.471	11.0 (2.5)	10.7 (2.7)	0.543
Area of BB on non-dominant side, cm^2^	8.9 (2.0)	8.1 (3.7)	0.412	6.5 (1.7)	6.2 (1.5)	0.372
SWV of BB on non-dominant side, m/s	2.0 (0.7)	2.2 (1.1)	0.890	2.4 (1.2)	2.6 (0.9)	0.923
Thickness of BB on dominant side, cm^a^	0.08 (0.02)	0.07 (0.02)	0.045	0.07 (0.02)	0.06 (0.01)	0.132
Perimeter of BB on dominant side, cm^a^	0.51 (0.11)	0.54 (0.10)	0.940	0.41 (0.18)	0.38 (0.08)	0.235
Area of BB on dominant side, cm^2^a^^	0.36 (0.07)	0.33 (0.10)	0.181	0.25 (0.07)	0.23 (0.06)	0.121
Thickness of BB on non-dominant side, cm^a^	0.08 (0.02)	0.07 (0.02)	**0** **.** **007**	0.07 (0.02)	0.06 (0.02)	0.460
Perimeter of BB on non-dominant side, cm^a^	0.49 (0.10)	0.53 (0.20)	0.411	0.39 (0.08)	0.38 (0.06)	0.453
Area of BB on non-dominant side, cm^2^a^^	0.34 (0.10)	0.30 (0.15)	0.700	0.24 (0.08)	0.23 (0.07)	0.399

The data in bold represent statistically significant differences. BMI, body mass index.

a Muscle dimension parameters (thickness, perimeter, area) were normalized by dividing by BMI.

There was no significant difference in ultrasound measurements between MCI and non-MCI groups, sub grouped by gender (Table 3).

**Table 3:** Difference of ultrasound measurement for MCI and non-MCI group with subgroup analysis by gender.

Parameter	Male			Female		
	Non-MCI (*n* = 16)	MCI (*n* = 11)	*P* value	Non-MCI (*n* = 54)	MCI (*n* = 40)	*P* value
Thickness of BB on dominant side, cm	2.1 (0.5)	2.1 (0.5)	0.881	1.7 (0.2)	1.7 (0.4)	0.260
Perimeter of BB on dominant side, cm	12.9 (2.9)	13.6 (3.6)	0.256	10.9 (2.2)	10.7 (1.7)	0.435
Area of BB on dominant side, cm^2^	8.9 (2.1)	9.6 (2.6)	0.226	6.4 (1.9)	6.6 (2.3)	0.963
SWV of BB on dominant side, m/s	2.7 (1.4)	2.3 (0.9)	0.348	2.5 (1.5)	2.8 (1.1)	0.381
Thickness of BB on non-dominant side, cm	2.0 (0.7)	2.0 (0.7)	0.766	1.7 (0.2)	1.8 (0.4)	0.141
Perimeter of BB on non-dominant side, cm	12.0 (3.0)	12.9 (2.8)	0.348	10.7 (2.6)	10.6 (1.6)	0.359
Area of BB on non-dominant side, cm^2^	8.3 (3.3)	8.4 (2.0)	0.489	6.4 (1.8)	6.2 (1.6)	0.930
SWV of BB on non-dominant side, m/s	3.1 (1.4)	2.1 (0.8)	0.203	2.6 (1.2)	2.9 (1.0)	0.165
Thickness of BB on dominant side, cm^a^	0.08 (0.02)	0.08 (0.03)	0.921	0.06 (0.01)	0.06 (0.01)	0.677
Perimeter of BB on dominant side, cm^a^	0.51 (0.14)	0.53 (0.12)	0.278	0.40 (0.10)	0.38 (0.09)	0.066
Area of BB on dominant side, cm^2^a^^	0.35 (0.06)	0.35 (0.09)	0.30	0.24 (0.06)	0.23 (0.08)	0.278
Thickness of BB on non-dominant side, cm^a^	0.08 (0.01)	0.08 (0.03)	0.844	0.06 (0.02)	0.07 (0.02)	0.789
Perimeter of BB on non-dominant side, cm^a^	0.48 (0.14)	0.52 (0.09)	0.554	0.39 (0.09)	0.38 (0.09)	0.090
Area of BB on non-dominant side, cm^2^a^^	0.38 (0.10)	0.35 (0.13)	0.537	0.23 (0.08)	0.23 (0.07)	0.494

BMI, body mass index.

a Muscle dimension parameters (thickness, perimeter, area) were normalized by dividing by BMI.

The AUCs of BB thickness in predicting prefrailty among male patients ranged from 0.741 to 0.824, with the highest AUC observed on the non-dominant side. The corresponding optimal cut-off value was 1.85 cm, yielding a sensitivity of 77.8% and a specificity of 66.7%. Given that this analysis was based on only 9 prefrail male patients, the wide 95% CI (0.627–1.000) indicates substantial uncertainty, and these results should be interpreted with caution. For female participants, no parameters revealed a significant difference between the non-prefrail and prefrail groups. Consequently, no further analysis using ROC curves to predict prefrailty was conducted.

## Discussion

Prefrailty and mild cognitive impairment (MCI) are well-established risk factors associated with increased morbidity and mortality after surgery. However, the use of ultrasound (US) for preoperative identification of these conditions remains under-investigated. To address this gap, our exploratory pilot study aimed to explore the potential value of US and elastography measurements in predicting prefrailty. Notably, prefrailty and frailty show a stronger association with low skeletal muscle mass (sarcopenia) compared to MCI (8, 9). Previous research has demonstrated the promise of US measurements of quadriceps thickness in discriminating frail from non-frail surgical patients. Furthermore, these measurements have also been linked to adverse outcomes, such as prolonged hospitalization, increased morbidity, and non-home discharge (31, 33). However, our study population specifically focused on patients with hip or knee osteoarthritis, a condition that can affect lower limb muscle mass. Consequently, we opted to measure bilateral BB muscle dimensions, as they are less likely to be directly impacted by osteoarthritis in the lower limbs. Since there were inherent differences in BB muscle properties between males and females (32), all statistical analyses were adjusted with gender and BMI.

Consistent with prior research, our study found that male patients with prefrailty exhibited reduced muscle thickness. However, in contrast to studies solely focused on discriminating between frail and non-frail participants, our study found limited discriminatory ability of the US method in distinguishing prefrailty from non-prefrailty within this population, the AUC of the best US indicator were found to be 0.824, with a wide 95% CI (0.627–1.000) reflecting the small sample size of the male subgroup. A potential explanation for the discrepancy can be attributed to our study design. We exclusively recruited patients, whereas the previous study included both patients and volunteers. The inherent health differences between patients and healthy volunteers are likely to be more pronounced than variations within a patient population. This could explain why US effectively identified reduced muscle thickness in our prefrail patients, while encountering challenges in distinguishing prefrail from non-prefrail individuals in a clinical setting.

No parameters revealed a significant difference between the non-prefrail and prefrail groups for female participants. The observed difference in the predictive value of BB dimension for prefrailty between males and females may be attributed to several potential factors: First, males and females exhibit inherent biological differences in muscle composition, distribution and function due to variations in hormonal levels (34, 35), which may influence how BB dimension correlates with prefrailty. Second, as males generally have greater baseline muscle mass and larger BB diameters compared to females, this difference in muscle size could result in a stronger association between BB measurements and prefrailty in males, while the smaller range of variation in females might limit the predictive ability. Third, the mechanisms and rates of muscle loss (sarcopenia) differ from gender. Males may experience more pronounced changes in specific muscle groups, including the BB (36, 37), which could make these measurements more sensitive predictors of prefrailty in males than in females. Additionally, factors such as differential patterns of physical activity, fat infiltration into muscle (myosteatosis), or gender-specific phenotypic expressions of frailty may also contribute to the observed disparity.

Besides muscle quantity and dimension, frailty and sarcopenia are characterized by declines in muscle quality. Shear wave elastography (SWE), by measuring muscle stiffness, offers promise for detecting these conditions due to its ability to assess muscle quality alongside traditional measures of muscle size (16–18). Janczyk et al. conducted a systematic review to evaluate the diagnostic utility of sonoelastography for sarcopenia (18). Nine studies employed shear wave elastography (SWE), and one utilized one-strain elastography. However, due to significant heterogeneity among the included studies, the authors were unable to draw definitive conclusions regarding the effectiveness of elastography in assessing sarcopenia. We aimed to determine whether SWE could also serve as a potential tool for identifying prefrailty patients, given the significant association between sarcopenia and frailty syndrome (38). We hypothesized that there would be a difference in muscle stiffness between the prefrail and non-prefrail groups; however, our analysis did not reveal any statistically significant differences. One possible explanation is age-related myosteatosis (fatty infiltration), which affects shear wave propagation and may confound SWE measurements, particularly in older adults. This was not directly assessed in our study. The lack of significant differences in SWE measurements between prefrail and non-prefrail groups suggests that muscle stiffness may not be as sensitive as muscle thickness for early prefrailty detection. This may reflect that muscle quality deteriorates later than muscle mass in the aging process. Technical factors such as probe positioning, muscle anisotropy, and contraction state may also influence SWE reliability, particularly in older adults with varying muscle composition.

In addition to investigating prefrailty, we explored the potential of US to predict MCI in this patient population undergoing total hip or knee replacement surgery. In this study, there was no significant difference of ultrasound measurement between MCI and non-MCI groups, suggesting that grayscale US and SWE may not be reliable tools for predicting or differentiating MCI from non-MCI in this context. This null finding is biologically plausible given the indirect link between upper limb muscle morphology and cognitive function. While bedside point-of-care ultrasound (POCUS)—such as thoracic and lung US—has shown success in predicting acute neurocognitive decline (delirium) by detecting acute somatic stressors like respiratory or circulatory failure, static peripheral muscle dimensions appear less sensitive to chronic, stable subclinical cognitive impairments like MCI (39, 40). Future research on preoperative cognitive risk stratification might benefit from more direct assessments, such as neuroimaging or biomarkers of neurodegeneration.

Our study had a few limitations. First, we selected only patients who underwent total hip/knee replacement surgeries, thereby excluding those who received other types of surgeries. This exclusion introduces a selection bias, limiting the generalizability of our findings to the broader patient population undergoing various surgical procedures. Second, our investigation did not encompass the assessment of the impact of US measurements on secondary outcomes, such as unplanned ICU admission and hospital stay duration. The omission of secondary outcomes precludes a comprehensive evaluation of the effectiveness and value of US in predicting prefrailty. Third, the proposed cutoff value (1.85 cm) was derived from a small sample of prefrail males (*n* = 9) in a single-center study, limiting its reliability and precluding immediate clinical application. This cutoff requires validation in independent, adequately powered cohorts. Due to the limited sample size, particularly in the male subgroup, we were unable to perform multivariable logistic regression adjusting for potential confounders such as age, BMI, and nutritional status. Future studies with larger samples are needed to determine whether BB thickness provides independent predictive value beyond these covariates. Furthermore, standardized, population-specific reference values remain to be established. This is particularly relevant since pre-frailty and frailty are major predisposing factors for postoperative delirium, a highly prevalent and severe complication widely documented in geriatric patients undergoing major orthopedic procedures, such as hip fracture surgeries, and associated with poor institutional outcomes (41–43). Fourth, the FRAIL scale was chosen for its practicality and speed in a busy preoperative setting. However, as a self-reported instrument, it may be subject to differential misclassification across genders due to potential differences in self-perception of fatigue, activity limitations, and illness perception. In our cohort, the proportion of females was significantly higher in the prefrail group (86.2% vs. 67.9% in males, *P* = 0.016). This raises the possibility that male prefrailty classification on the FRAIL scale may correlate more strongly with objective physical muscle loss, whereas female prefrailty may capture a broader or different phenotypic construct. Future studies should incorporate objective physical performance measures, such as handgrip strength, to complement self-reported scales. Fifth, SWE measurements in the contracted state have limited methodological precedent, and the reproducibility of this specific protocol remains less established than that of relaxed-state measurements.

## Conclusions

In this pilot study, preoperative US measurements of BB thickness demonstrated preliminary, hypothesis-generating promise for predicting prefrailty among male patients undergoing total hip/knee replacement. However, the proposed cut-off value derived from a small male subgroup requires validation in larger, independent cohorts before clinical translation. This approach showed limited utility in female participants and was not effective in predicting MCI.

## Data Availability

Raw data supporting the conclusions of this article are available from the corresponding authors upon reasonable request.
